# *Helicobacter pylori* Protein JHP0290 Binds to Multiple Cell Types and Induces Macrophage Apoptosis via Tumor Necrosis Factor (TNF)-Dependent and Independent Pathways

**DOI:** 10.1371/journal.pone.0077872

**Published:** 2013-11-01

**Authors:** Sushil Kumar Pathak, Raquel Tavares, Nele de Klerk, Anna-Lena Spetz, Ann-Beth Jonsson

**Affiliations:** 1 Department of Molecular Biosciences, The Wenner-Gren Institute, Stockholm University, Stockholm, Sweden; 2 Department of Medicine, Center for Infectious Medicine, Karolinska Institutet, Karolinska University Hospital Huddinge, Stockholm, Sweden; Emory University, United States of America

## Abstract

Activated macrophages at the sub-mucosal space play a major role in generating innate immune responses during *H. pylori* infection. Final disease outcome largely depends on how *H. pylori* and bacterium-derived products modulate macrophage responses. Here, we report that JHP0290, a functionally unknown protein from *H. pylori*, regulates macrophage functions. Recombinant purified JHP0290 (rJHP0290) had the ability to bind to several cell types including macrophages, human gastric epithelial cell lines, human monocyte-derived dendritic cells (MoDC) and human neutrophils. Exposure to rJHP0290 induced apoptosis in macrophages concurrent with release of proinflammatory cytokine tumor necrosis factor (TNF). A mutant strain of *H. pylori* disrupted in the *jhp0290* gene was significantly impaired in its ability to induce apoptosis and TNF in macrophages confirming the role of endogenous protein in regulating macrophage responses. Intracellular signaling involving Src family of tyrosine kinases (SFKs) and ERK MAPK were required for rJHP0290-induced TNF release and apoptosis in macrophages. Furthermore, rJHP0290-induced TNF release was partly dependent on activation of nuclear transcription factor-κB (NF-κB). Neutralizing antibodies against TNF partially blocked rJHP0290-induced macrophage apoptosis indicating TNF-independent pathways were also involved. These results provide mechanistic insight into the potential role of the protein JHP0290 during *H. pylori*-associated disease development. By virtue of its ability to induce TNF, an acid suppressive proinflammatory cytokine and induction of macrophage apoptosis, JHP0290 possibly helps in persistent survival of the bacterium inside the stomach.

## Introduction

*H. pylori* is a Gram-negative microaerophilic bacterium that selectively colonizes human gastric and duodenal mucosa [1]. Most infections are asymptomatic and persistent infection can cause chronic gastritis that may lead to development of gastroduodenal ulcers, gastric adenocarcinoma and gastric MALT lymphoma [1]. Infection induces strong innate and adaptive immune responses, but in most cases this fails to eradicate the bacterium. *H. pylori* has generally been considered as a non-invasive pathogen, however, several studies have shown that *H. pylori* itself and bacterium-derived products can invade the gastric mucosa and remain in direct contact with immune cells of lamina propria [2]–[4]. Macrophages form essential components of innate immune responses against *H. pylori*. However, the bacterium is able to protect itself by strategies that limit effector functions of the macrophages. Studies have shown that *H. pylori* prevents phagocytosis by macrophages and also induces apoptosis in macrophages [5]–[9]. *H. pylori* induces macrophage apoptosis by polyamine-dependent mechanisms and signaling via ERK MAPK-dependent formation of the activator protein-1 (AP-1) complex is involved [6], [7], [10].

*H. pylori* infection is associated with the induction of various chemokines and cytokines including IL-8, TNF, IL-6 and IL1β which play an important role in ultimate disease outcome [1]. IL1β and TNF are acid-suppressive proinflammatory cytokines, which are significantly increased within *H. pylori*-colonized human gastric mucosa [11]–[13]. Polymorphisms which increase IL1β and TNF expression have been associated with an increased risk of gastric cancer and its precursors [14], [15]. Studies have indicated that activated macrophages are a major source of above chemokines and cytokines during *H. pylori* infection [16]–[20]. *H. pylori* proteins HP0175 and HP0986 have been shown to interact with macrophages *in vitro* via Toll-like receptor 4 (TLR4) and Tumor necrosis factor receptor-1, respectively [17], [21]. Recombinant HP0986 induces apoptosis and release of IL-8 and TNF from macrophages concurrent to the activation of the key transcription factor NF-κB [17]. HP0175 induces IL6 release from macrophages via activation of mitogen-and stress-activated protein kinase-1 and NF-κB [18]. Other *H. pylori* proteins such as vacuolating cytotoxin A (Vac A), Urease and JHP0940 have also been shown to activate macrophages [16], [22], [23].

Studies have identified many *H. pylori* virulence associated molecules such as cytotoxin-associated gene A, Vac A, adhesins, several other effectors and toxins [1]. Although their functional role has been suggested in various studies, associations of many known virulence factors with different disease outcomes have contradicting evidences. For example, studies have indicated that clinical course of infection does not correlate with presence or absence of the best studied virulence factors cytotoxin-associated gene A and Vac A in the Oriental population [24], [25], suggesting the involvement of additional factors in disease development, which are still unidentified. There are several hypothetical and unknown proteins coded by the *H. pylori* genome whose functional role in pathogenesis is unexplored or poorly defined. Therefore, it is pertinent to look into the biology of novel genes/proteins to get new insight into pathogenesis. Considering the general noninvasive nature of *H. pylori*, it is believed that proteins which are released by the bacterium are playing a major role in the disease development. With this in view, we focused on the secreted protein HP0305 [26]. HP0305 is highly conserved among different *H. pylori* strains. The homolog of HP0305 in the *H. pylori* strain J99 (used in this study) was identified as JHP0290 [27]. Using a proteomic approach, Olofsson et al. have demonstrated the presence of HP0305 in outer membrane vesicles, which are considered as a delivery vehicle for the transport of virulence factors from the bacterium to the target cells [28], [29]. Another study has reported overexpression of HP0305 under acidic stress condition, an environment encountered by the bacterium inside the human stomach [30]. HP0305 is strongly recognized by the sera of *H. pylori* infected patients and a recent study has further suggested that HP0305 could be one of the potential biomarker for gastric cancer risk in China [31], [32]. In addition, HP0305 contains a domain of homology to the regulators of G protein signaling, suggesting that protein HP0305 might have an effect on the G protein transmitted signaling pathway of the host cell.

In this study, we have explored the possible role of JHP0290 during *H. pylori* pathogenesis. We provide evidence that rJHP0290 binds to several target cell types. In addition, rJHP0290 induces apoptosis and TNF in macrophages. Functional inactivation of the *jhp0290* gene in *H. pylori* strain J99 led to significantly reduced ability to induce apoptosis and TNF in macrophages. Furthermore, we have identified some of the signaling pathways and molecules regulating rJHP0290-induced apoptosis and TNF in macrophages.

## Results

### Expression of JHP0290 in *H. pylori* Strain J99

**Figure 1:**
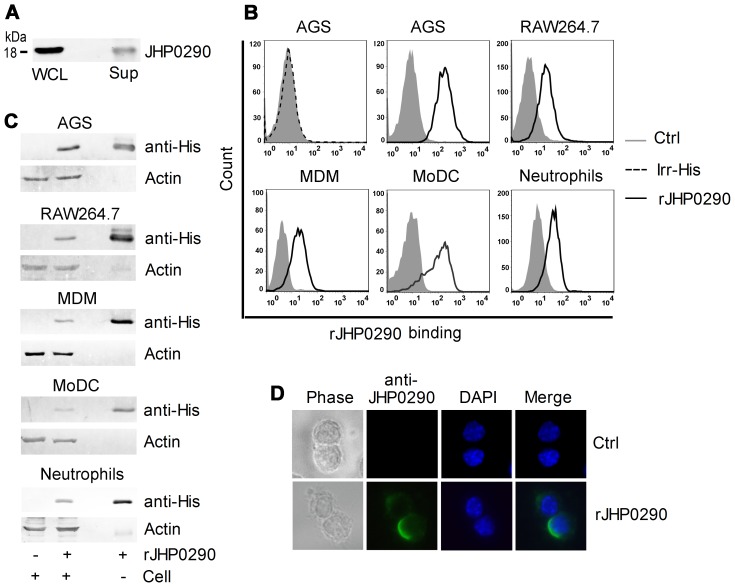
Expression of JHP0290 in *H. pylori* strain J99 and binding of rJHP0290 to mammalian cells. (**A**) Whole cell lysate (WCL) and culture medium (Sup) of *H. pylori* strains J99 were immunoblotted with anti-JHP0290 antibody. (**B**) Different cells as indicated in figure legends were treated either with protein storage buffer (Ctrl) or with rJHP0290 (2 µg/ml) for 15 min. AGS cells were incubated with an irrelevant His-tagged protein (Irr-His, 2 µg/ml) for 15 min. Cells were washed, stained with anti-His-tag antibody and Alexa Fluor 647 conjugated secondary antibody followed by flow cytometry analysis. (**C**) Cells were incubated with rJHP0290 as described above followed by washing of unbound protein with PBS. Cell lysates were prepared in SDS-PAGE sample buffer. Cell lysates and purified denatured rJHP0290 were immunoblotted with anti-His-tag antibody. Blots were reprobed with anti-actin antibody. (**D**) RAW264.7 cells were treated with rJHP0290 as described above. Immunofluorescence analysis was performed using an antibody raised against rJHP0290 and Alexa Fluor 488-labeled secondary antibody. Cells were mounted in ProLong® Gold mounting medium with DAPI. One representative experiment of three is shown.

Sequence homology analysis indicated that JHP0290 is highly conserved among *H. pylori* strains. We first verified expression of the protein in *H. pylori* strain J99 by immunoblotting using an antibody raised against rJHP0290. Expression of JHP0290 was observed in J99 (Fig. 1A). JHP0290 was also detected in the growth medium of *H. pylori* (Fig. 1A) indicating that the protein is released by the bacterium during growth, which is in agreement with the observations by Kim et al [26].

### rJHP0290 Binds to Multiple Cell Types

Proteins, which are released by bacteria, often exert their effect by binding to certain receptors on cells. Previous studies have identified several such proteins from *H. pylori* [1]. Considering the release of JHP0290 by *H. pylori* during growth [26], [28], we explored the possible binding of the purified protein to various target cells such as epithelial cells, macrophages, dendritic cells and neutrophils. In order to study the binding of rJHP0290, cells were incubated with His-tagged rJHP0290, followed by staining with anti-His-tag antibody and appropriate Alexa Fluor-647 conjugated secondary antibody. By FACS we could detect binding of rJHP0290 to a human gastric epithelial cell line (AGS), a murine macrophage cell line (RAW264.7), primary human monocyte-derived macrophages (MDM), MoDC and human neutrophils (Fig. 1B). In order to rule out the nonspecific effects of His-tag present in recombinant protein, an irrelevant His-tagged protein (Irr-His) was used in parallel. We could not detect binding of Irr-His to AGS cells (Fig. 1B) and other cell types (data not shown) confirming the specific interaction of rJHP0290 with the cell types tested. Binding of rJHP0290 to above cell types was also detected by immunoblotting with anti-His-tag antibody (Fig. 1C). In addition to above cell types, we could also detect binding of rJHP0290 to PMA treated human monocytic cell line (THP-1) and another gastric epithelial cell line (MKN45) (data not shown). rJHP0290 binding assays by FACS and immunoblotting were also performed using anti-JHP0290 antibody which gave similar results (data not shown). We further confirmed the binding of rJHP0290 to macrophages by immunofluorescence staining using anti-JHP0290 antibody (Fig. 1D). Taken together, these results indicated that rJHP0290 is recognized by multiple cell types.

### rJHP0290 Induces Macrophage Apoptosis and Release of TNF from Macrophages

**Figure 2:**
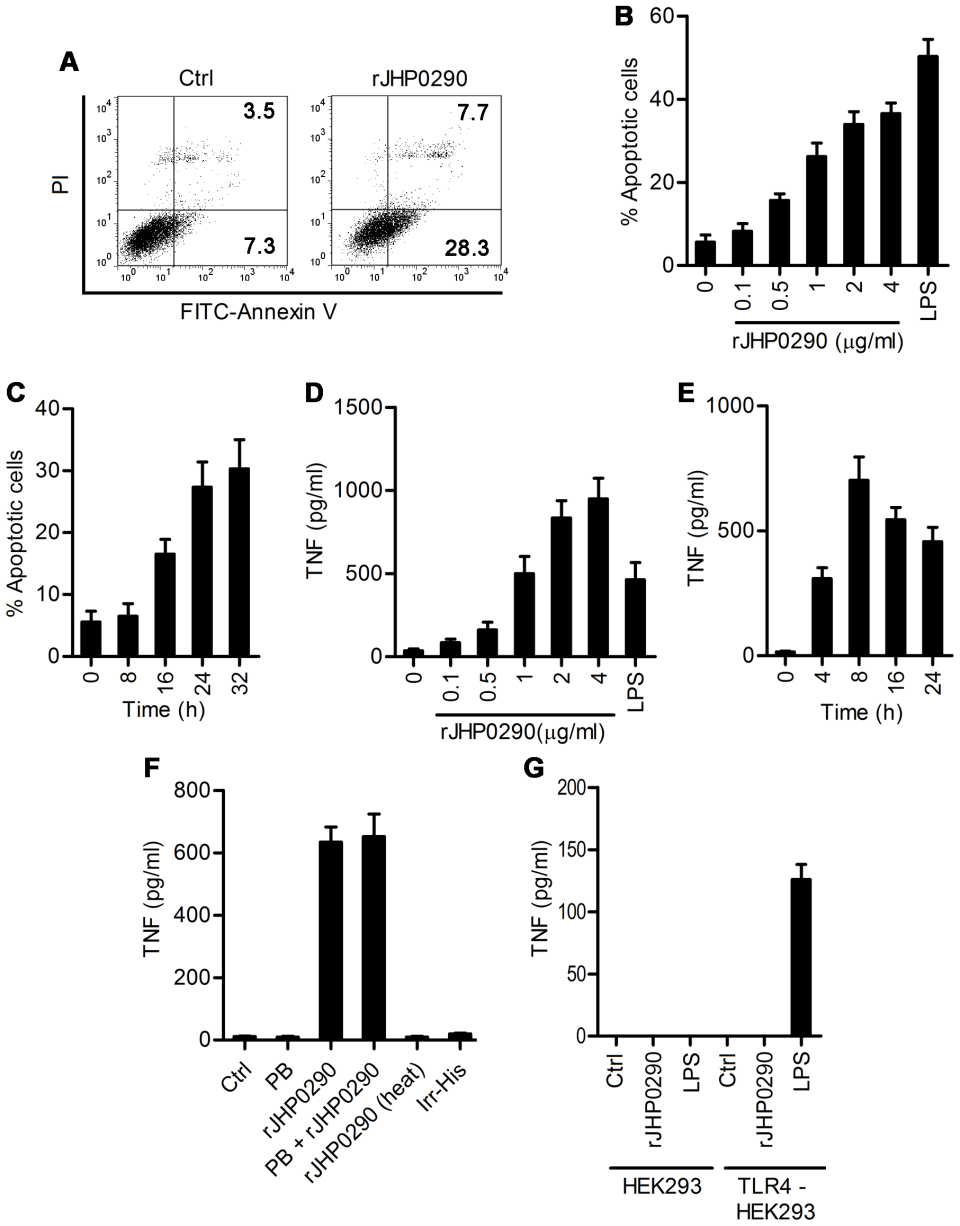
rJHP0290 induces apoptosis and TNF in macrophages. (**A**) RAW 264.7 cells were incubated either with protein storage buffer (Ctrl) or rJHP0290 (2 µg/ml) for 24 h. Cells were washed, stained with FITC-conjugated Annexin V antibody and propidium iodide (PI) as described in material and methods and analyzed by flow cytometry. RAW264.7 cells were treated with various concentrations of rJHP0290 as indicated in figure legends (**B**) or treated with rJHP0290 (2 µg/ml) for different time periods (**C**). Apoptotic cells were analyzed by flow cytometry. RAW264.7 cells were treated with various concentrations of rJHP0290 as indicated in figure legends (**D**) or treated with rJHP0290 (2 µg/ml) for different time periods (**E**). TNF level in the conditioned medium was measured by ELISA. (**F**) rJHP0290 was subjected to boiling for 30 min (heat) or was treated with polymyxin B (PB) for 1 h before treatment of RAW264.7 cells for 16 h. TNF level in the conditioned medium was measured by ELISA. (**G**) HEK293 or HEK293-hTLR4A-MD2-CD14 (TLR4-HEK293) cells were treated with either rJHP0290 (2 µg/ml) or *E. coli* LPS (500 ng/ml) for 16 h. TNF level in the conditioned medium was measured by ELISA. Values indicate mean ± SD of three independent experiments.

Cytokine and chemokine production is strongly up-regulated during *H. pylori* infection and certain virulence factors from *H. pylori* induce apoptosis in epithelial cells and macrophages, which has a major impact on ultimate disease outcome. Considering the ability of rJHP0290 to bind to epithelial cells and macrophages, we further explored the possible induction of apoptosis and proinflammatory cytokines. Cells were exposed to rJHP0290 and induction of apoptosis was determined by FACS after staining with Annexin V and propidium iodide (PI). Representative flow cytometry analysis of apoptosis is depicted in Fig. 2A. rJHP0290 was found to induce apoptosis in RAW264.7 cells in a dose- and time-dependent manner (Fig. 2B and 2C). Under similar conditions, rJHP0290 did not induce significant apoptosis in AGS and MKN45 cells (data not shown). In addition to induction of apoptosis, rJHP0290 stimulated the release of proinflammatory cytokine TNF from RAW264.7 cells in a dose- and time-dependent manner (Fig. 2D and 2E). Heat treatment inhibited the TNF-inducing ability of rJHP0290 (Fig. 2F), supporting the view that TNF induction was due to protein rJHP0290. Irr-His protein failed to induce TNF release from RAW264.7 cells ruling out the possibility of the His-tag contributing to the observed effect (Fig. 2F). The LPS antagonist Polymyxin B (PB) treatment had no effect on rJHP0290-induced TNF release (Fig. 2F), ruling out the possibility of the observed induction of TNF being due to LPS contamination. In addition, PB treatment had no effect on rJHP0290-induced apoptosis in RAW264.7 cells (Figure S1A). To further confirm that the observed effect was not due to LPS contamination, studies were performed in HEK293 cells, which are naturally deficient in receptors for binding and signaling by LPS and HEK293-hTLR4A-MD2-CD14 cells stably transfected with LPS signaling receptors TLR4/MD2/CD14. We could not detect any significant induction of TNF after treating HEK293 or HEK293-hTLR4A-MD2-CD14 cells with rJHP0290 (Fig. 2G). As expected, our positive control *Escherichia coli* LPS induced TNF release from HEK293-hTLR4A-MD2-CD14 cells but not in HEK293 cells (Fig. 2G). These results indicated that the observed effect was not due to LPS contamination in the purified protein. It was interesting to note that rJHP0290 had the ability to bind to both HEK293 and HEK293-hTLR4A-MD2-CD14 cells also (data not shown), suggesting the involvement of receptors other than TLR4/MD2/CD14.

**Figure 3:**
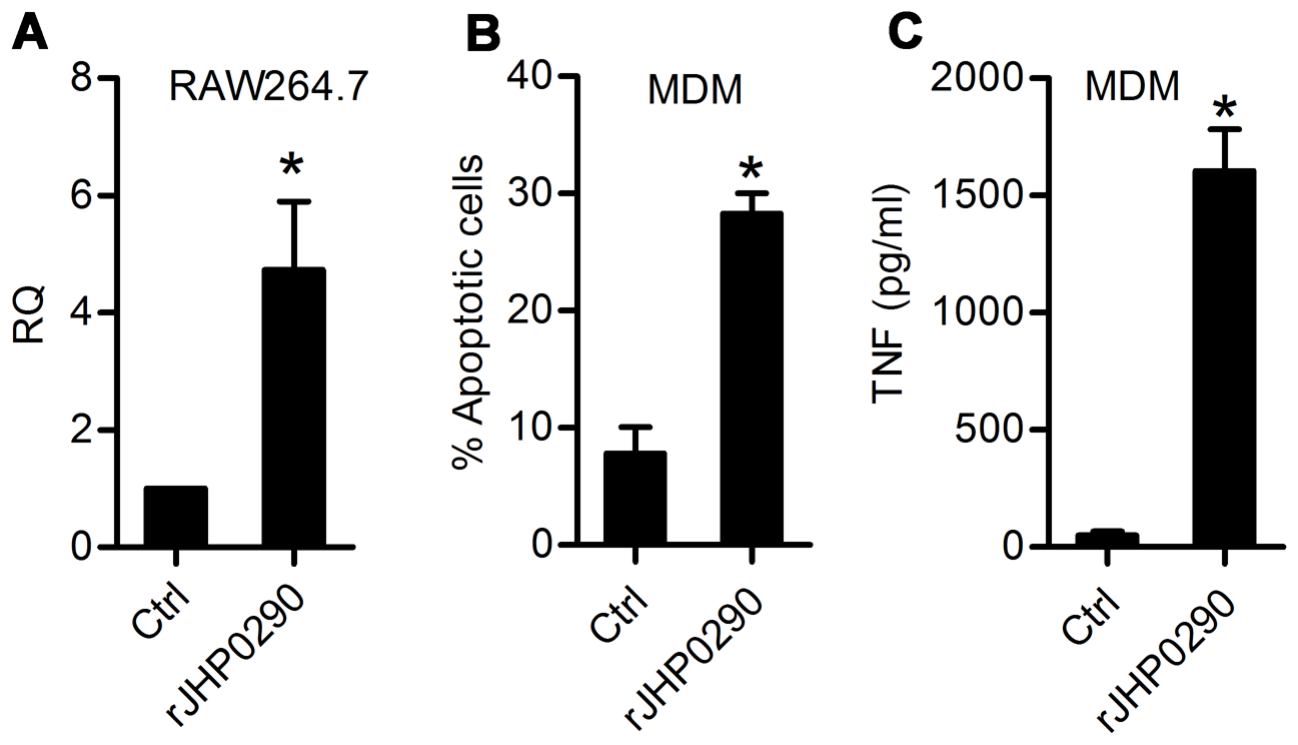
TNF mRNA expression in RAW264.7 cells and apoptosis and TNF induction in primary MDM after rJHP0290 treatment. (**A**) Real-time PCR was used to determine fold changes (RQ) in mRNA of TNF after treatment of RAW264.7 cells with rJHP0290 (2 µg/ml) for 6 h. Monocyte derived macrophages (MDM) were treated either with protein storage buffer (Ctrl) or rJHP0290. Percentage of apoptotic cells (**B**) or TNF release in culture supernatant (**C**) was determined. Values indicate mean ± SD of three independent experiments. Statistically significant differences are indicated by *(p<0.05).

To determine whether the effect of rJHP0290 on TNF release was attributed to regulation at the level of TNF gene transcription, TNF mRNA level was assessed in control untreated and rJHP0290 treated macrophages by quantitative real-time PCR. We could detect more than 5-fold upregulation of TNF mRNA in RAW 264.7 cells after exposure to rJHP0290 (Fig. 3A). Apoptosis and TNF inducing ability of rJHP0290 was further assessed in primary human MDM. Significantly higher apoptosis and TNF induction in MDM was observed after rJHP0290 treatment (Fig. 3B and 3C).

### *H. pylori* Deficient in JHP0290 Protein is Impaired in Apoptosis and TNF-inducing Ability in Macrophages

**Figure 4:**
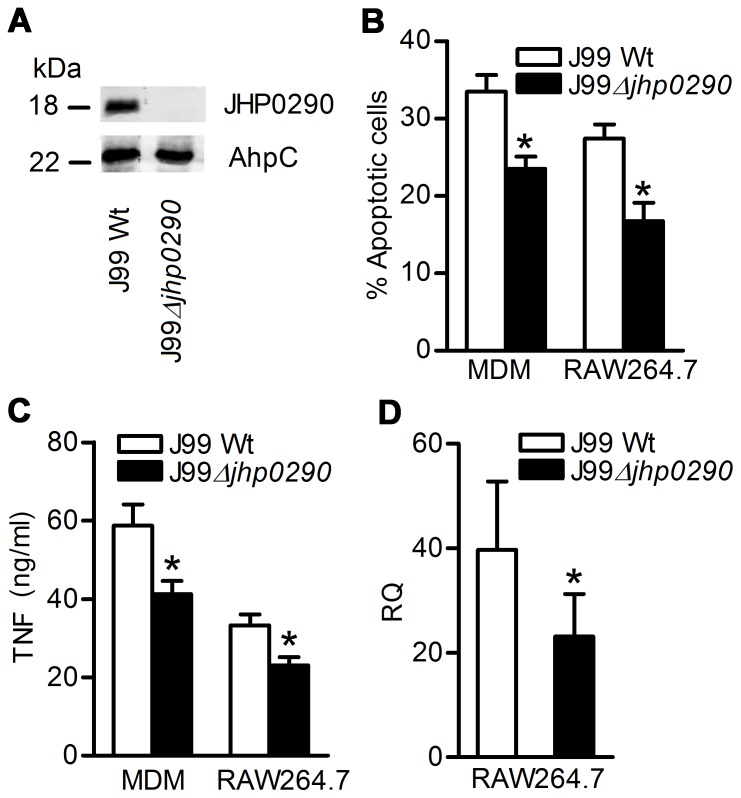
The role of endogenous JHP0290 on macrophage apoptosis and TNF release. (**A**) Equal amount of cell lysates from *H. pylori* wild-type strain (J99 Wt) or mutant strains (J99 Δ*jhp0290*) were immunoblotted with anti-JHP0290 antibody. Blots were reprobed with antibody raised against another *H. pylori* protein, alkyl hydroperoxide-reductase (AhpC), to confirm equal loading. Blot shown is representative of results obtained in three independent experiments. MDM and RAW264.7 cells were infected either with *H. pylori* wild-type strain J99 (Wt) or mutant strain (Δ*jhp0290*) at MOI100. Percentage of apoptotic cells (**B**) or TNF release in culture supernatant (**C**) was determined. (**D**) RAW264.7 cells were infected either with J99 Wt or J99 Δ*jhp0290* mutant strain at MOI100 for 6 h. RT-PCR was performed to assess the fold changes (RQ) in mRNA level of TNF. Values indicate mean ± SD of three independent experiments. Statistically significant differences are indicated by *(p<0.05).

In order to study the apoptosis and TNF-inducing ability of endogenous JHP0290, an isogenic mutant strain of *H. pylori* J99 disrupted in the *jhp0290* gene was constructed. Inactivation of the *jhp0290* gene in mutant strain was verified by PCR and immunoblotting with anti-JHP0290 antibody (Fig. 4A). Inactivation of the *jhp0290* gene led to a significant reduction in the induction of apoptosis (Fig. 4B) and TNF release (Fig. 4C) compared with the wild-type parent strain both in RAW264.7 and primary MDM. The impaired TNF inducing ability of the mutant strain was also observed at mRNA level.(Fig. 4D). However, the apoptosis and TNF-inducing ability was not totally abolished in the mutant strain. This was expected because several other apoptosis and TNF-inducing factors from *H. pylori* also contribute to apoptosis and TNF release from macrophages [16], [17], [22]. Taken together, these results suggested the involvement of JHP0290 in the induction of apoptosis and TNF in macrophages.

### rJHP0290-induced Apoptosis is Dependent on TNF and Caspases

**Figure 5:**
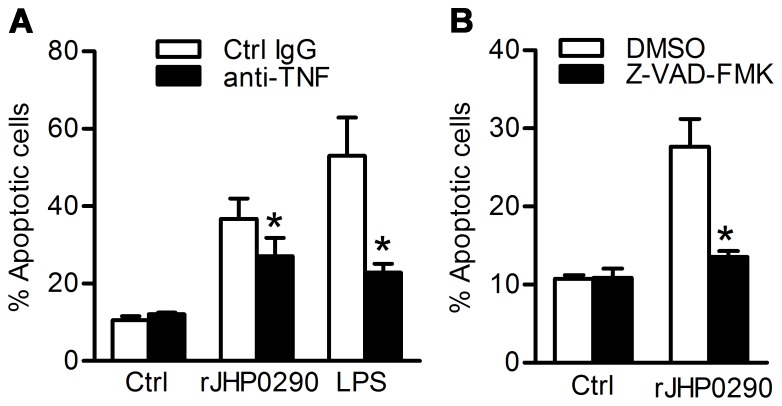
The role of TNF and caspases on rJHP0290-induced macrophage apoptosis. (**A**) RAW264.7 cells were pretreated either with isotype control IgG or TNF neutralizing antibody (10 µg/ml) for 1 h followed by treatment with either rJHP0290 (2 µg/ml) or LPS (500 ng/ml) for 24 h. Apoptotic cells were analyzed by staining with AnnexinV antibody and PI using Flow cytometry. (**B**) RAW264.7 cells were treated either with DMSO or caspase inhibitor Z-VAD-FMK (20 µM) for 1 h followed by treatment with rJHP0290 (2 µg/ml) for 24 h. Percentage of apoptotic cells was determined. Values indicate mean ± SD of three independent experiments. Statistically significant differences are indicated by *(p<0.05).

Considering that induction of TNF has in many instances been related to the triggering of apoptotic signaling, we investigated whether rJHP0290-induced apoptosis in macrophages was linked to TNF. rJHP0290-induced apoptosis in RAW264.7 was significantly inhibited in the presence of anti-TNF neutralizing antibody (Fig. 5A). However, the blocking effect appeared partial with 25.7±5.9% inhibition at the highest of concentration of TNF neutralizing antibody (10 µg/ml). In case of LPS treated cells, >60% inhibition was observed even at lower concentration of the TNF neutralizing antibody (2.5 µg/ml) indicating that the blocking antibody used in the study was effective in blocking TNF. Collectively, these results suggested that TNF independent signaling pathways were also involved in r-JHP0290-induced apoptosis in RAW264.7 cells. In addition, a Pan-caspase inhibitor zVAD-FMK significantly blocked rJHP0290-induced apoptosis in RAW264.7 cells (Fig. 5B) indicating that rJHP0290 triggered caspase dependent apoptosis.

### SFKs and ERK MAPK are Involved in TNF Induction and Macrophage Apoptosis by rJHP0290

**Figure 6:**
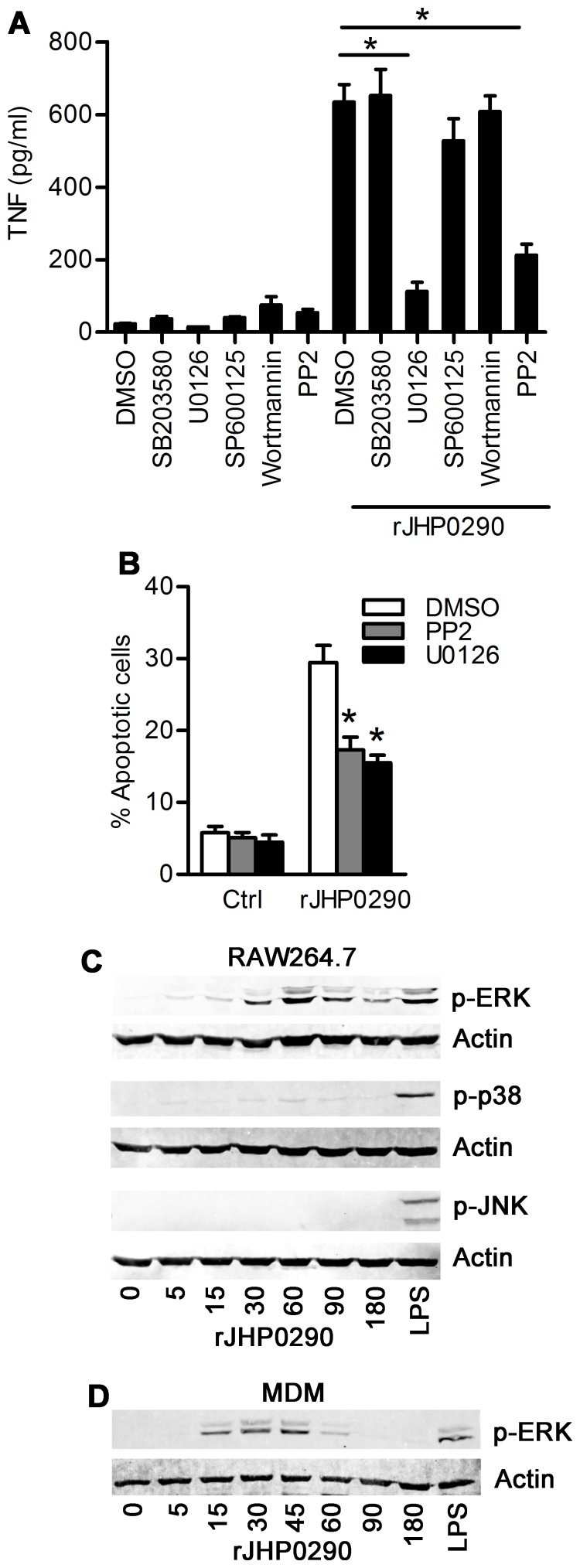
The role of SFKs and ERK MAPK on rJHP0290 mediated responses in macrophages. RAW264.7 cells were pretreated with vehicle control or different inhibitors as indicated in the figure legends followed by treatment with rJHP0290 (2 µg/ml). TNF release in culture supernatant (**A**) or percentage of apoptotic cells (**B**) was determined. Values indicate mean ± SD of three independent experiments. Statistically significant differences are indicated by *(p<0.05). (**C**) RAW264.7 cells were treated with rJHP0290 for various time points (min) as indicated in figure legends. Positive controls cells were treated with *E. coli* LPS for 30 min. Cell lysates were prepared and immunoblotted with anti-phospho-ERK, anti-phospho-p38 and anti-phospho-JNK antibody. All blots were reprobed with anti-actin antibody to confirm equal loading. Blots shown are representative of results obtained in three independent experiments. (**D**) MDMs were treated with rJHP0290 for various time points (min) as indicated in figure legends. Cell lysates were prepared and immunoblotted with anti-phospho-ERK antibody followed by reprobing with anti-actin antibody to confirm equal loading. Blot shown is representative of results obtained in three independent experiments.

Although, *H. pylori* and bacterium-derived proteins have been shown to induce TNF release from macrophages [16], [17], the signaling pathways involved have not been studied well. Therefore, we reasoned that it would be worthwhile to explore the signaling pathways involved in rJHP0290-induced TNF release from macrophages. Cells were pre-treated with specific inhibitors to block activation or activity of the target signaling molecule/pathways, followed by treatment with rJHP0290 and the assessment of TNF level in the culture supernatant by ELISA. Blocking of SFKs and ERK MAPK using PP2 and U0126 respectively, significantly blocked rJHP0290-induced TNF release from RAW264.7 cells, whereas blocking of other pathways such as p38 MAPK by SB203580, JNK MAPK by SP600125 and PI3K by Wortmannin had no effect (Fig. 6A). As expected, rJHP0290 induced apoptosis was also significantly inhibited in the presence of U0126 and PP2 (Fig. 6B) whereas blocking of p38 MAPK and JNK MPAK had no effect (data not shown). We could detect a similar trend in the inhibitory effect of PP2 or U0126 pretreatment on rJHP0290-induced TNF release in MDM (data not shown). Collectively, these results suggested that rJHP0290-induced TNF release and macrophage apoptosis is regulated by ERK MAPK and SFKs.

Further, in order to verify the activation of ERK MAPK by rJHP0290 in macrophages, the level of ERK 1/2 phosphorylation was evaluated after treating cells with rJHP0290 for various periods of time. rJHP0290 elicited a time-dependent activation of ERK in RAW264.7 cells with maximum activation after 60 min of treatment (Fig. 6C). There was very weak activation of p38 MAPK, as assessed by level of phosphorylated p38 MAPK, under similar conditions (Fig. 6C). rJHP0290 failed to induce activation of JNK MAPK (Fig. 6C). We could also detect activation of ERK MAPK in primary MDM after treatment with rJHP0290 albeit with different kinetics with maximum activation after 30–45 min of rJHP0290 treatment (Fig. 6D).

### Transcription Factors Involved in TNF Induction by rJHP0290

**Figure 7:**
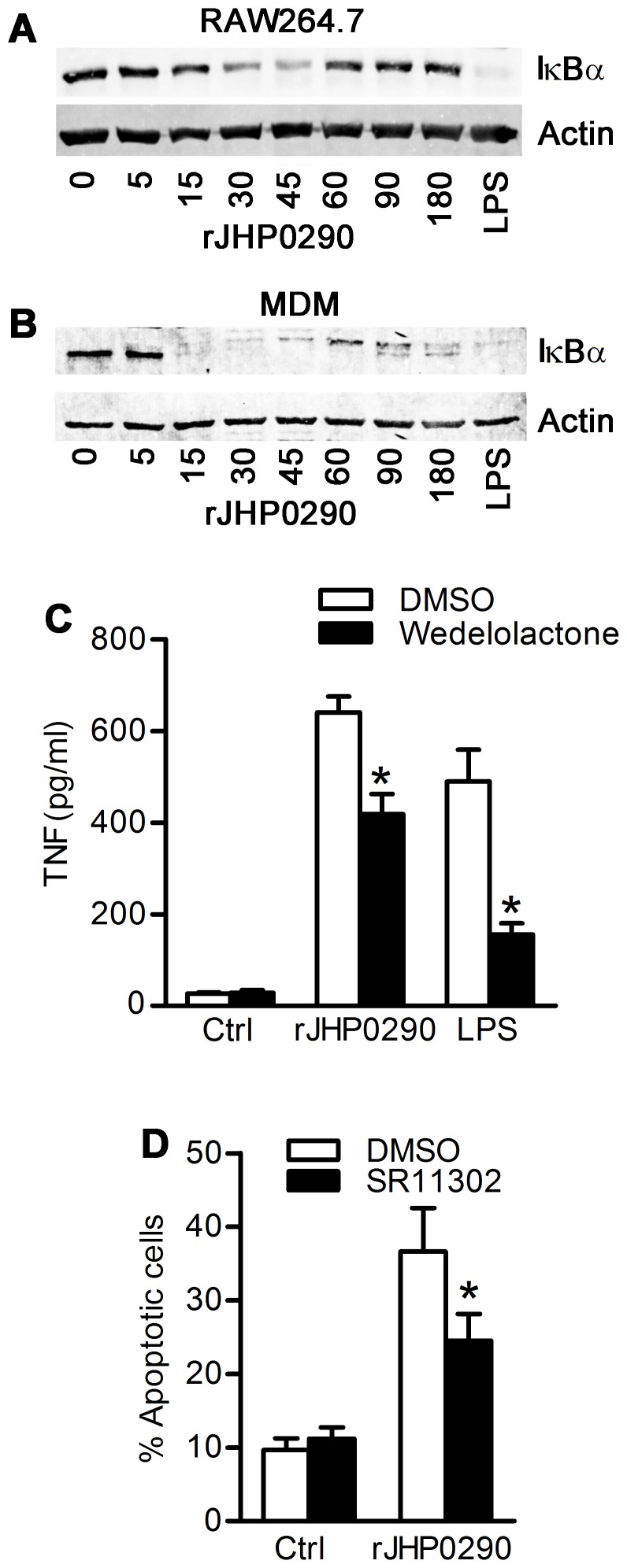
The role of NF-κB and AP-1 transcription factors in rJHP0290 mediated responses in macrophages. RAW264.7 cells (**A**) or MDM (**B**) were treated with rJHP0290 for various time points (min) as indicated in figure legends. Positive controls cells were treated with *E. coli* LPS for 30 min. Cell lysates were prepared and immunoblotted with anti-IκBα antibody followed by reprobing with anti-actin antibody to confirm equal loading. Blot shown are representative of results obtained in three independent experiments. RAW264.7 cells were treated either with DMSO or IKK inhibitor Wedelolactone (50 µM) or AP-1 inhibitor SR11302 (1 µM) for 1 h followed by treatment with rJHP0290. TNF release in culture supernatant (**C**) or percentage of apoptotic cells (**D**) was determined. Values indicate mean ± SD of three independent experiments. Statistically significant differences are indicated by *(p<0.05).

TNF induction is known to be regulated by several transcription factors with NF-κB being the most important regulator [33], [34]. First, we assessed the level of IκBα in cells after treatment with rJHP0290. We could detect degradation of IκBα, which is an indicator of NF-κB activation, in RAW264.7 (Fig. 7A) and primary MDM (Fig. 7B) after treatment with rJHP0290. In order to confirm that NF-κB activation is actually linked to rJHP290-induced TNF release, studies were performed using Wedelolactone, a chemical inhibitor which blocks activation of IKK kinase and therefore, phosphorylation and degradation of IκBα. Pre-treatment of RAW264.7 cells with Wedelolactone partially blocked rJHP0290-induced TNF release by 35.1±8.8% (Fig. 7C). However, in the case of LPS treated samples, the effect of Wedelolactone was more pronounced with 63.7±6.6% inhibition. These results suggested the involvement of additional transcription factors, apart from NF-κB, in regulation of rJHP0290-induced TNF release. This conclusion was further supported by the observation that rJHP0290-induced IκBα degradation was completely blocked by Wedelolactone (Figure S1B) confirming the inhibitory effect of the chemical at the concentration used in this study.

Several studies have identified the role of the AP-1 family of transcription factors in regulation of TNF induction in macrophages and the role of the AP-1 complex in regulation of macrophage apoptosis by *H. pylori* has also been reported [6], [35]. Therefore, we assessed the possible involvement of the AP-1 family of transcription factors in rJHP0290-induced apoptosis and TNF induction using AP1 inhibitor SR11302. Pretreatment of cells with SR11302 had no effect on rJHP0290-induced TNF induction from macrophages (data not shown). However, SR11302 could significantly block rJHP0290-induced apoptosis in macrophages (Fig. 7D). Collectively, these results indicated that the AP-1 family of transcription factors regulates rJHP0290-induced apoptosis in macrophages, whereas rJHP0290-induced TNF release is independent of the AP-1 activation.

## Discussion

Chronic *H. pylori* infection is characterized by infiltration of mononuclear cells to the lamina propria accompanied with increased expression of proinflammatory cytokines and chemokines. Presence of *H. pylori* and bacterium-derived products has been observed in the lamina propria *in vivo* [36]. Considering the critical role of macrophages in mounting host immune responses against *H. pylori*, it is of obvious importance to identify the effectors of *H. pylori* that modulate macrophage responses to gain insight into events, which ultimately determine the final disease outcome. The association of currently known virulence factors of *H. pylori* with final disease outcome has contradicting observations making it important to identify the role of new potential virulence factors in disease development [24], [25]. The present study is a step toward this end with focus on the protein JHP0290 and macrophage responses.

In this study, we have demonstrated that JHP0290 regulates macrophage responses during *H. pylori* infection. We observed binding of rJHP0290 to macrophage cell lines and primary human MDM. In addition to binding to macrophages, the protein had the ability to bind to several other cell types including gastric epithelial cell lines, MoDCs and neutrophils, suggesting that the protein binds to certain cell surface receptor(s) which is/are present on multiple cell types and is possibly involved in regulation of responses in those cell types. Currently, we are performing studies to identify the cell surface receptor involved in recognition of rJHP0290. Our preliminary results are indicating that TLRs requiring Myd88 as an adaptor molecule are probably not involved in recognition of rJHP0290 since silencing of Myd88 had no effect on rJHP0290-induced TNF induction from macrophages (unpublished observation). However, further studies are necessary before drawing any conclusions. We are also exploring the responses of other cell types to rJHP0290.

rJHP0290 challenged RAW264.7 cells and MDM underwent apoptosis in a dose- and time-dependent manner. However, the same protein failed to induce apoptosis in gastric epithelial cell lines AGS and MKN45 under similar conditions. These results indicated that JHP0290 may have cell type-specific effects. Considering previous reports indicating that the observed effect of recombinant proteins purified from *E. coli* could be due to contamination with endotoxin from bacteria, extreme care was taken to rule out this possibility. The purified protein was incubated with endotoxin removal column and endotoxin content in the elute was measured. We also performed studies in HEK293 null cells, which lack receptors for LPS signaling, and HEK293-hTLR4A-MD2-CD14 cells stably transfected with the LPS signaling receptors TLR4/CD14/MD2. rJHP0290 bound similarly to both cell types without significant induction of TNF, further ruling out the possibility of endotoxin contamination. In addition it was interesting to note that rJHP0290 did not induce release of another cytokine IL-1β from RAW264.7 cells under similar conditions (unpublished observation). These observations confirmed rJHP0290 specific effects in the current experimental system. This view was further strengthened by our observation that a mutant of *H. pylori* strain J99 disrupted in the *jhp0290* gene was significantly impaired in its ability to induce apoptosis and TNF in macrophages.

TNF is an acid-suppressive proinflammatory cytokine and studies have shown that the level of TNF is increased within *H. pylori* colonized human gastric mucosa [11], [12]. Polymorphisms in TNF leading to increased expression have been linked to increased risk of gastric cancers [14]. Using transgenic mice that overexpress β-catenin agonist Wnt1, Oguma et al. observed that *H. pylori* infection lead to rapid infiltration of macrophages within dysplastic mucosa in close apposition to gastric epithelial cells and suggested that macrophage derived TNF promotes Wnt/β-catenin signaling, which may contribute to tumor development in gastric mucosa [37]. Therefore, by induction of TNF from activated macrophages, JHP0290 can potentially contribute to gastric cancer development. Previous reports showing the strong recognition of JHP0290 homolog by sera of *H. pylori* infected patients and the association of seropositivity to the protein with increased risk for gastric cancer further indicates a possible link between expression of JHP0290 and gastric cancer development. Studies have also shown that initial infiltration of macrophages in the *H. pylori* infected gastric mucosa is followed by gradual loss during persistent infection [38]. As suggested by Asim et al., this gradual loss was likely due to induction of apoptosis [6]. The clearance of macrophages is probably necessary to protect bacteria from phagocytic and antibacterial activities of macrophages. By induction of apoptosis in macrophages during prolonged infection, JHP0290 may help in survival of the bacterium in the gastric mucosa. A balance between activation of macrophages and their suppression by apoptosis is the key to survival of the bacterium and subsequent disease development.

Studies have shown that *H. pylori* infection induces activation of NF-κB and AP-1 family of transcription factors, which are involved in regulation of various cytokines and chemokines [17], [39], [40]. *H. pylori* proteins JHP0940 and HP0986 have been shown to induce TNF from macrophages concurrent to the nuclear translocation of NF-κB [16], [17]. However, in those studies it was not confirmed whether observed activation of NF-κB is actually linked to TNF release. It is possible that the observed NF-κB activation is actually involved in regulation of other cellular processes. To address this possibility, we have used an IKK kinase inhibitor (Wedelolactone), which inhibits NF-κB activation by blocking phosphorylation and subsequent degradation of the NF-κB inhibitory protein IκBα and showed that the observed activation of NF-κB was actually linked to TNF release since pretreatment of cells with Wedelolactone significantly blocked rJHP0290-induced TNF induction from macrophages. Furthermore, we have characterized the upstream signaling pathways involved in the induction of TNF by rJHP0290. Several signaling pathways and transcription factors are known to regulate TNF induction in macrophages. However, in case of *H. pylori* infection-induced TNF in macrophages, signaling pathways are not well characterized. Our results indicated that signaling via ERK MAPK and SFKs are involved in rJHP0290-induced TNF release from macrophages. To our knowledge, this is the first report of a role for ERK MAPK and SFKs in the induction of TNF from macrophages by a secreted antigen of *H. pylori*. In addition to regulation of TNF release from macrophages, ERK MAPK and SFKs also regulated rJHP0290-induced macrophage apoptosis. This regulation was partly via induction of TNF since neutralizing antibodies against TNF could partially block rJHP0290-induced macrophage apoptosis. TNF exists in soluble and trans membrane forms. Studies have shown that both forms are involved in regulation of macrophage apoptosis [41], [42]. Blocking of soluble TNF by neutralizing antibodies prevents its binding to TNF receptors on macrophages and subsequent downstream signaling [41]. Ligation of neutralizing antibodies with trans membrane TNF may induce reverse signaling leading to elimination of the activated monocytes and macrophages [43]. Further studies are required to understand how differential blocking of soluble and trans membrane TNF may affect rJHP0290-induced macrophage apoptosis.

In addition, it appears that TNF independent apoptosis regulation is also involved which is likely via activation of the AP-1 family of transcription factors since AP-1 inhibitor SR11302 could significantly block rJHP0290-induced macrophage apoptosis without affecting TNF release. Recently, Asim et al. have shown that *H. pylori* induced macrophage apoptosis is regulated by the AP-1 protein complex [6]. However, *H. pylori* virulence factor(s) specifically regulating AP-1 dependent macrophage apoptosis remained unidentified. Our results are indicating that JHP0290 is one of the factors from *H. pylori,* which regulate AP-1 dependent macrophage apoptosis. Further studies are required to identify the role of JHP0290 in regulation of the AP-1 complex components, c-Myc and Ornithine decarboxylase, which are involved in regulation of macrophage apoptosis during *H. pylori* infection.

In summary, in this report we have provided functional insight into the potential role of JHP0290, a previously uncharacterized hypothetical protein from *H. pylori,* during interaction with macrophages. These findings are important in context to *H. pylori* infection considering that the disease progression is the outcome of complex events during interaction of known and potential virulence factors with target immune cells. A detailed understanding of the role played by each of them is critical for the development of strategies aimed at controlling infections leading to more serious disease outcomes.

## Materials and Methods

### Bacterial Strains and Culture Conditions

*H. pylori* strain J99 (ATCC 700392) was grown on Columbia blood agar plates (Acumedia) supplemented with 8% horse blood and 8% horse serum at 37°C under microaerophilic conditions. For liquid culture, cells were grown in Brucella broth (Acumedia) containing 8% horse serum with shaking at 37°C under microaerophilic conditions. *E. coli* BL21 (DE3) and *E. coli* DH5α strains were grown in Luria-Bertani Miller (LBM) media.

### Cell Culture and Treatments

All cell lines were obtained from American Type Culture Collection, except for the HEK293-hTLR4A-MD2-CD14 and MKN45 (JCRB0254) cells, which were from InvivoGen and Japan Health Science Research Resource Bank, respectively. The human gastric epithelial cell lines AGS (CRL-1739), MKN45 and human monocytic cell line THP-1 (TIB-202) were maintained in RPMI-1640 (Invitrogen) with 10% heat-inactivated fetal calf serum (FCS) (Invitrogen). THP-1 cells were differentiated into macrophages using 100 nM PMA (Sigma) for 3 days. The murine macrophage cell line RAW264.7 (TIB-71) was cultured in DMEM (Invitrogen) with 10% FCS. HEK293 cells were cultured in DMEM with 10% FCS, 50 U/ml penicillin, 50 µg/ml streptomycin, and 2 mM l-glutamine. HEK293-hTLR4A-MD2-CD14 cells were cultured in the same growth medium supplemented with 10 µg/ml Blasticidin (InvivoGen) and 50 µg/ml HygroGold (InvivoGen). Above cell lines were maintained in serum-containing growth medium without any antibiotics during the treatments described below. As indicated, cells were treated with 0.1–4 µg/ml of purified rJHP0290 or the same volume of protein storage buffer. TLR4 ligands *Escherichia coli* LPS (500 ng/ml; Sigma-Aldrich) was used as a positive control. For studies with inhibitors, cells were pretreated with either vehicle alone or various inhibitors as indicated for 30–60 min, followed by the addition of only protein storage buffer or rJHP290. All inhibitors were purchased from (Calbiochem, Merck4biosciences) except the AP1 inhibitor SR11302, which was from R&D Systems. Mouse anti-TNF neutralizing antibody (2.5–10 µg/ml, clone MP6-XT22; MAB4101) was purchased from R&D Systems. For infection studies, cells were incubated with wild-type *H. pylori* strain J99 (J99 Wt) or *jhp0290*-deficient mutant strain (J99 *Δjhp0290*) at MOI100 for various periods of time.

### *In vitro* Differentiation of MDMs and MoDCs

Buffy coats from healthy human blood donors were obtained from the blood bank at Karolinska University Hospital Huddinge. CD14^+^ monocytes were enriched from buffy coats by negative selection using RosetteSep human monocyte enrichment cocktail (Stem Cell Technologies) followed by separation using a Ficoll density gradient (Stem Cell Technologies). Purity of monocytes was assessed by staining with anti-CD14^+^ antibody (Clone TUK4; DAKO). Monocytes were further enriched through plastic adherence for 2 h followed by washing of unbound cells. Plate bound monocytes were cultured for 6–7 days in RPMI 1640 medium supplemented with 10% FCS and recombinant human macrophage colony-stimulating factor (M-CSF; 50 ng/ml; Immunotools) to obtain immature MDMs. Differentiation of monocytes into macrophages was assessed morphologically by light microscopy and staining with antibodies against CD68 (clone Y1/82A; BD biosciences) and CD14 surface markers, followed by flow cytometry using LSRFortessa flow cytometer (BD Biosciences). For generation of MoDCs, purified monocytes were incubated in complete medium supplemented with recombinant human cytokines IL-4 (6.5 ng/ml; R&D Systems) and granulocyte-macrophage colony-stimulating factor (GM-CSF; 250 ng/ml; Peprotech) for 6–7 days to obtain immature DCs. Differentiation of monocytes into DCs was assessed by staining with antibodies against CD14, CD1a (Clone NA1/34; DAKO), CD80 (Clone 307.4; BD Biosciences) and CD86 (Clone 2331/FUN-1; BD Biosciences) surface markers by flow cytometry.

### Generation of *H. pylori* J99*Δjhp0290* Mutant Stain

The Chloramphenicol resistance gene (CAT) was amplified by PCR from the pACY184 vector using primer pair Cat 1 (5′-GGGTTTTTGCAAAAATCAGTAAGTTGGCAGCATCAC-3′) and Cat 2 (5′- CGACCAAGTCAACTTATTATCACTTATTCAGGCGTA-3′). *H. pylori* genomic DNA was isolated from bacteria grown for 48 h using a Genomic DNA isolation kit (Promega) as per manufacturer’s instructions. Two pairs of *jhp0290*-specific primers A (5′- AGAACGGGTATGATTTGTGGGGTAGT-3′), B (5′- ACTTACTGATTTTTGCAAAAACCCTGCTAGTAAAAC-3′) and C (5′- AGTGATAATAAGTTGACTTGGTCGCAAGTGGAAATC -3′), D (5′-AGCCACTAAAGTGTGTTTGCTAAAATCAT -3′), were used to PCR amplify the up- and downstream regions, respectively. PCR products were purified using the Gel purification kit (GE healthcare). The primers were designed with overlapping homologies at the ends. The resulting three PCR products were combined into one fragment by five cycles without any primers followed by 30 cycles with primers A and D. The PCR product was introduced into *H. pylori* by natural transformation using the method of spot transformation. Bacteria grown for 48 h were suspended in Brucella broth and spotted as 100 µl (from 1×10^8^ bacteria/ml stock) onto Columbia blood agar plates. After incubation with 150 ng of purified DNA product for 6 h, the bacterial transformation spot was spread over the plate and incubated for further 24 h. The bacteria were then moved onto selective Columbia blood agar plates containing 15 µg/ml of Chloramphenicol and incubated for 3 to 7 days. Positive clones were confirmed by PCR. Lack of JHP0290 expression in the mutant strain was also verified by immunoblotting using an antibody raised against rJHP0290.

### *H. pylori* Protein Extraction and Immunoblotting with Anti-JHP0290 Antibody

For preparation of whole cell lysates of *H. pylori*, 1×10^8^ cells were mixed with 100 µl of SDS-PAGE sample reducing buffer (45 mM Tris-Cl pH 6.8, 10% Glycerol, 1% SDS and 5% β-mercaptoethanol) and boiled at 95°C for 10 min. *H. pylori* culture supernatant was run through 0.45 µM-pore size filter (Millipore) before addition of SDS-PAGE sample reducing buffer. 10 µl of cell lysates and 30 µl of culture supernatants were separated on 12.5% SDS-polyacrylamide gels and transferred electrophoretically to polyvinylidene difluoride membranes (Millipore). The blots were blocked with 5% (w/v) non-fat dry milk (NFDM) in PBS containing 0.1% (v/v) Tween 20 (PBST) for 1 h and subsequently incubated overnight at 4°C with anti-JHP0290 antibody in blocking buffer. Polyclonal antibody against JHP0290 was generated by EZbiolab, USA followed by Protein A affinity purification. After washing with PBST, membranes were incubated with appropriate secondary antibody conjugated to Odyssey IR-dye (Li-COR) for 1 h. Membranes were visualized using an Odyssey IR scanner (Li-COR) at standardized 700 and 800 nm intensity settings.

### Cloning, Expression and Purification of JHP0290

Using the genomic DNA from *H. pylori* J99 as template, the construct for expression of rJHP0290 was generated by cloning of the PCR product at NdeI and XhoI sites of the vector pET28b^+^ (Novagen). Plasmid construct was transformed into *E. coli* BL21 (DE3). Induction of the protein was conducted at 37°C for 2–3 h with isopropyl thio-β-d-galactoside (100 µM). Hexa-His-tagged JHP0290 was purified from the cell lysates by chromatography on Talon resin (Clontech). Purified protein was extensively dialyzed overnight to remove imidazole and upon fractionation on 12% polyacrylamide gel, the protein showed a single band on staining with Coomasie brilliant blue dye. Dialyzed protein was further incubated with endotoxin removal column (Pierce) and endotoxin content of the purified protein was measured by endotoxin detection kit (Pierce) with detection limit of 0.1 EU/ml.

### Binding Assay for rJHP0290 by Immunoblotting and FACS

Cells were treated with rJHP0290 (2 µg/ml) for 15 min followed by extensive washing with PBS to remove unbound protein. For Immunoblotting with anti-His-tag antibody (Millipore), whole cell lysates were prepared in SDS-PAGE sample reducing buffer by heating at 95°C for 10 min. For studying binding of rJHP0290 by FACS, cells were incubated with anti-His-tag antibody (1∶1000 dilution) in FACS buffer (2% BSA in PBS) for 1 h on ice followed by washing with FACS buffer and incubation with Alexa 647 conjugated anti-mouse IgG antibody (Molecular probes,1∶5000 dilution) in FACS buffer for 30 min on ice. After incubation, cells were washed twice with FACS buffer and analyzed by LSRFortessa flow cytometer.

### Binding Assay for rJHP0290 by Immunofluorescence

13 mm glass cover slips were coated with poly-D-lysine (5 µg/ml in PBS). RAW264.7 cells were grown onto these overnight, reaching approximately 50% confluence. The cells were then treated with 2–5 µg/ml of rJHP0290 for 15 min. Unbound protein was removed by washing in PBS. The cover slips were incubated in blocking buffer (2% BSA, 0.1% Tween20 in PBS) for 30 min in room temperature. Cells were then incubated with anti-JHP0290 antibody (1∶5000 dilution) for 1 h at room temperature. Cells were washed with blocking buffer and incubated with Alexa Fluor 488-labeled secondary antibody (1∶5000 dilution) for 1 h at room temperature. The cover slips were fastened onto object glasses using ProLong® Gold mounting medium with DAPI (4′,6′-diamidino-2-phenylidole) (Invitrogen). Images were taken with an Axiovision Cell Observer HS fluorescent microscope (Carl Zeiss).

### Measurements of Apoptosis

Cells were stained with FITC-conjugated Annexin V antibody (BD Biosciences) and PI (BD Biosciences) following manufacturer’s instructions. Briefly, 1×10^5^ cells in 100 µl of Annexin binding buffer (10 mM HEPES pH 7.4, 140 mM NaCl, and 2.5 mM CaCl_2_) were mixed with 1 µl of FITC-conjugated Annexin V antibody. The mixture was incubated for 15 min at room temperature in dark and analyzed by flow cytometry. PI (2.5 µl) was added to the cell suspension just before analysis. The relative number of cells that were Annexin V-positive and/or PI positive was determined.

### ELISA for TNF

Conditioned medium was collected after various periods of time and assayed for TNF by ELISA using the TNF assay kit (Invitrogen and Biolegend) following the manufacturer’s instructions.

### RNA Isolation and Real Time PCR

RNA was isolated using the RNeasy minikit (Qiagen). 750 ng of total RNA was used for cDNA synthesis using Superscript Vilo CDNA synthesis kit (Fermentas) in a final reaction volume of 20 µl. Real-time PCR was performed with 0.5 µl cDNA using the LightCycler® 480 SYBR green I master (Roche) in a LightCycler® 480 Real-Time PCR System (Roche Diagnostics). Forward and reverse primers 5′-TCCCAGGTTCTCTTCAAGGGA-3′ and 5′-TCCCAGGTTCTCTTCAAGGGA-3′, respectively, were used for amplification of murine TNF. Glyceraldehyde-3-phosphate dehydrogenase (GAPDH) was used as a reference gene. GAPDH was amplified using primer pairs 5′-CATGGCCTTCCGTGTTCCTA-3′ (Forward) and 5′-GCGGCACGTCAGATCCA-3′ (Reverse). Data analysis was performed with the LightCycler® 480 Software 1.5 using the comparative cycle threshold method, in which target mRNA is normalized to the reference gene. The specificity was checked by analyzing the melting curves. Data are presented as fold changes in mRNA copy number in the treated cells as compared with mRNA in cells cultured in medium only.

### Preparation of Mammalian Cell Lysates and Immunoblotting

After treatments, cells were washed twice with PBS. Cell lysates were prepared by addition of appropriate amount of SDS-PAGE sample reducing buffer to the cells, followed by heating at 95°C for 10 min. Immunoblotting was performed using anti-phospho ERK1/2, anti-phospho-p38, anti-phospho-JNK and anti-IκBα alpha antibodies from Cell Signaling Technology. Anti-Actin antibody (Millipore) was used to confirm equal loading.

### Statistical Analysis

Student’s t-test was used for statistical analysis. A *p* value of <0.05 was considered statistically significant.

## Supporting Information

Figure S1 **Effect of Polymyxin B (PB) and Wedelolactone on rJHP0290-induced responses in macrophages. (A)** rJHP0290 or *E. coli* LPS was incubated with PB for 1 h before treatment of RAW264.7 cells for 24 h. Percentage of apoptotic cells was determined by staining with FITC-conjugated Annexin V antibody and PI followed by flow cytometry. Values indicate mean ± SD of three independent experiments. Statistically significant difference is indicated by *(p<0.05). **(B)** RAW264.7 cells were pretreated either with DMSO or IKK inhibitor Wedelolactone (50 µM) for 1 h followed by treatment with rJHP0290 for 30 min. Cell lysates were prepared and immunoblotted with anti-IκBα antibody followed by reprobing with anti-actin antibody to confirm equal loading. Blot shown is representative of results obtained in three independent experiments.
(TIF)
