# Supplementary figures and images for: Helicobacter pylori Protein JHP0290 Binds to Multiple Cell Types and Induces Macrophage Apoptosis via Tumor Necrosis Factor (TNF)-Dependent and Independent Pathways
Source: PLoS One. 2013 Nov 1;8(11):e77872. doi: 10.1371/journal.pone.0077872 (PMC3815203; doi:10.1371/journal.pone.0077872)

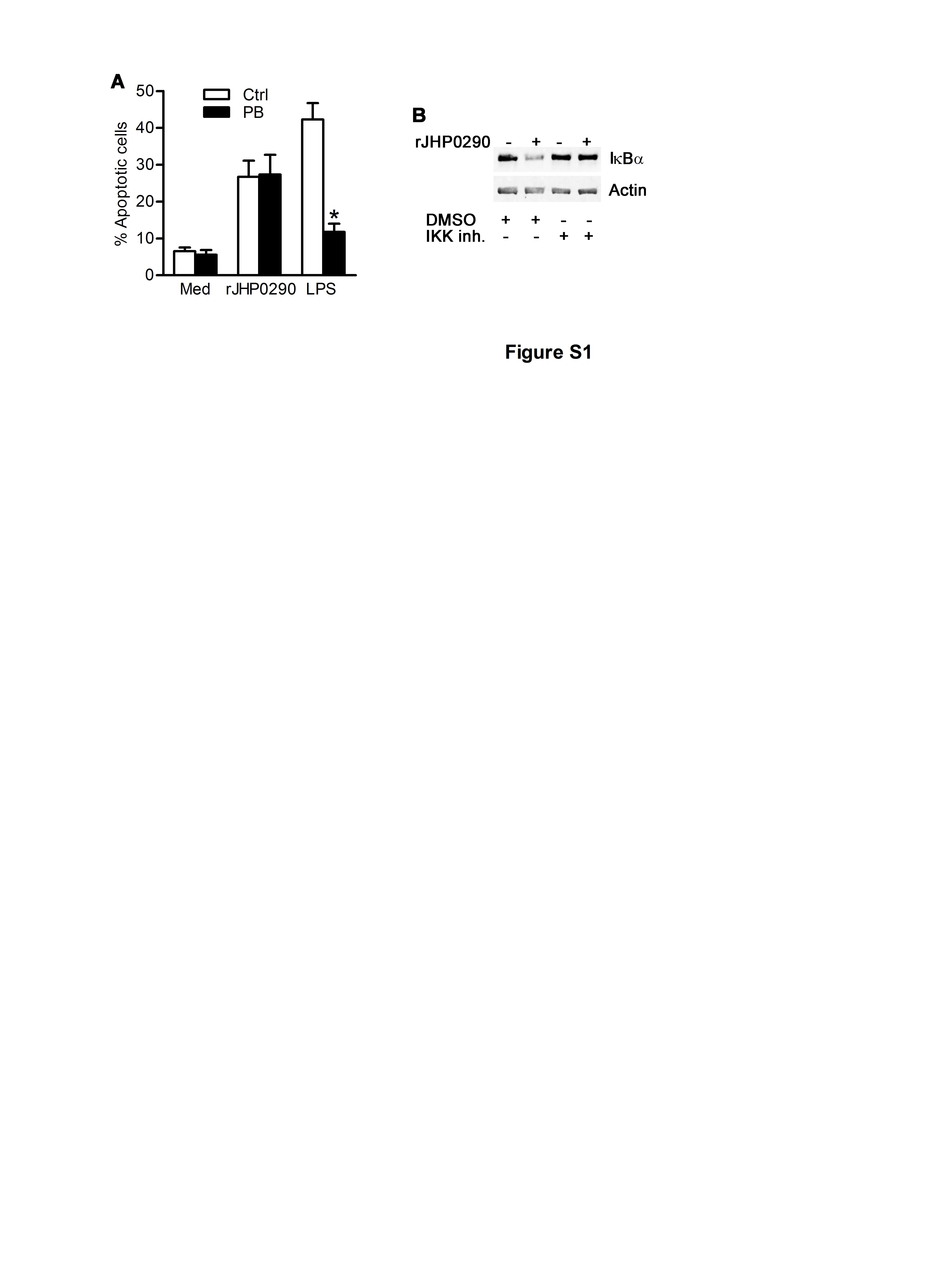

Supplement: Figure S1 — Effect of Polymyxin B (PB) and Wedelolactone on rJHP0290-induced responses in macrophages. (A) rJHP0290 or E. coli LPS was incubated with PB for 1 h before treatment of RAW264.7 cells for 24 h. Percentage of apoptotic cells was determined by staining with FITC-conjugated Annexin V antibody and PI followed by flow cytometry. Values indicate mean ± SD of three independent experiments. Statistically significant difference is indicated by *(p<0.05). (B) RAW264.7 cells were pretreated either with DMSO or IKK inhibitor Wedelolactone (50 µM) for 1 h followed by treatment with rJHP0290 for 30 min. Cell lysates were prepared and immunoblotted with anti-IκBα antibody followed by reprobing with anti-actin antibody to confirm equal loading. Blot shown is representative of results obtained in three independent experiments. (TIF) [file pone.0077872.s001.tif]
